# Effect of Reactivity of Hydrated Portland Cement on Hydrothermal Synthesis of Xonotlite

**DOI:** 10.3390/ma16041578

**Published:** 2023-02-14

**Authors:** Saixin Wang, Zheng Niu, Dongmei Jin, Jian He, Yuandong Mu, Guotian Ye

**Affiliations:** 1 Henan Key Laboratory of High Temperature Functional Ceramics, School of Materials Science and Engineering, Zhengzhou University, Zhengzhou 450001, China; 2 Luoyang Sanhe New Material Technology Co., Ltd., Luoyang 471900, China; 3 Jiangsu Jingxin New Material Co., Ltd., Yangzhou 225000, China

**Keywords:** xonotlite, calcined hydrated cement, hydrated cement, reactivity, hydrothermal synthesis

## Abstract

Significant interest in waste-cement recycling has been stimulated because of the high contents of calcium and silicon in waste cement. The reactivity of calcium and silicon in the raw material is one of the important factors for the hydrothermal synthesis of xonotlite. Therefore, the effect of the reactivity of calcium and silicon in the waste cement on the hydrothermal synthesis of xonotlite was studied in this paper. Portland cement that was hydrated for 6 months, with the aim of simulating the waste cement, was used for the first time as the calcium and silicon source in the hydrothermal synthesis of xonotlite. As calcination would raise the reactivity of the hydrated cement, the effect of calcination of the Portland hydrated cement on the hydrothermal synthesis of xonotlite was investigated. The hydrated cement was calcined at 900 °C, and the hydrothermal synthesis was carried out at 220 °C for different times. The phases of the hydrothermal products were analyzed by XRD and TG-DSC, and it was noted that the calcination of hydrated cement affected the formation rate of xonotlite. The content of xonotlite increased from 18% (synthesized with hydrated cement without calcination) to 74% (synthesized from hydrated cement with calcination at 900 °C) during a reaction time of 24 h. Furthermore, the micromorphologies of xonotlite using calcined and hydrated cement were compared and discussed from the perspective of the reactivity of the starting materials.

## 1. Introduction

Recycling construction waste is necessary to achieve the goals of reducing environmental pollution and sustainable development. As more and more construction waste is generated by the demolition of old buildings, the construction industry has started to reduce construction waste by improving the mechanical properties of concrete to prolong its service life [1,2] and recycling waste concrete to prepare other products.

In terms of construction waste, 65% is waste concrete [3]. In recent decades, the interest in reusing waste concrete as an aggregate source has increased [4,5,6]. Additionally, the process of recovering aggregate from waste concrete produces a fine powder, which is mainly composed of waste cement. Waste cement is the part of waste concrete with the highest economic cost and the greatest environmental burden. At present, the research on waste cement has made some progress. Waste cement after calcination is used to replace part of the cement clinker for the preparation of concrete or low-carbon cement mortars [7,8]. The hydration capacity and mechanical properties of recycled cementitious materials [9,10] have been improved and used to develop green ultra-high performance concrete [11]. Reusing waste cement is one of the effective ways to recycle waste concrete. As a result, attention has been paid to increasing the reuse methods of waste cement.

Waste cement is mainly composed of hydration products, which exist in the form of calcium silicate hydrate (C-S-H gel) and calcium hydroxide (Ca(OH)_2_), and the C-S-H gel constitutes about 60–70% of the fully hydrated cement paste [12]. Therefore, it also has been proposed that waste cement could be a potential calcium source to sequestrate CO_2_ and produce carbonate [13]. Additionally, waste cement exposed to high temperatures could exhibit rehydration ability and be used to produce recycled cement [14]. However, the calcination temperature would affect the reactive of waste cement. It was reported [15] that high temperature heating at 1100 °C transformed the C-S-H gel into wollastonite, which caused the hydrated cement to be hardly reactive. Xuan and Shui [14] mentioned that the hydrated cement calcined at 900 °C has higher f-CaO content and increased fineness, which means that hydrated cement calcined at 900 °C has high reactivity with water. These reports provide a new perspective on reusing waste cement through calcination at a proper temperature as an active calcium and silicon source for the synthesis of useful calcium silicate hydrate products.

Xonotlite is a calcium silicate hydrate with a chemical formula of Ca_6_Si_6_O_17_(OH)_2_ that generally can be synthesized through a hydrothermal process using high-purity siliceous and calcareous materials [16]. Xonotlite has been used as a main constituent for heat-insulating material because of its high decomposition temperature, which is about 800–1000 °C [17,18]. Due to the fibrous microscopic morphology of xonotlite, the material is also used to prepare porous β-wollastonite ceramics [19] and reinforcing reagents for bioactive composites [20], and for improving the compactness of oil-well cement [21].

Thus far, xonotlite has been synthesized using various kinds of calcium and silicon sources. Additionally, it has been found that the reactivity of the raw materials is crucial for the efficient production of xonotlite. Smalakys and Geng et al. [22,23,24] used SiO_2_ with different levels of reactivity as silicon sources to synthesize xonotlite. The results show that the reactivity of silicon sources affects the probability of the existence of amorphous phases, the sequence of the intermediate phases’ existence and the formation rate of crystalline during the synthesis of xonotlite. The study on the synthesis of pure xonotlite using carbide slag [25,26] indicated that the ultrasonic modification for different times (160 w, 1–3 h) and calcination at different temperatures (700–1000 °C) not only have an impact on the reactivity of carbide slag but also the morphology of xonotlite secondary particles. The research of Wang [27] showed that CaO obtained by calcining CaCO_3_ at 1000 °C had the highest effective reactivity, which was conducive to the formation of xonotlite fiber.

However, there are few reports regarding the synthesis of xonotlite using waste cement, and more importantly, it is unclear whether the waste cement activated by calcination at a proper temperature would influence the hydrothermal synthesis of xonotlite. In this work, the hydrated cement cured for 6 months at room temperature was used to simulate the waste cement. After drying and grinding, part of the hydrated cement was calcined at the proper temperature of 900 °C, to activate the calcium and silicon sources in hydrated cement according to the aforementioned related research. Then, the xonotlite was prepared by hydrothermal synthesis using calcined hydrated cement and hydrated cement. The effect of calcination of hydrated cement on the reactivity of calcium and silicon in the hydrated cement, and consequently, the effect on the formation rate and micromorphology of xonotlite, were investigated.

## 2. Materials and Methods

### 2.1. Materials

**Figure 1:**
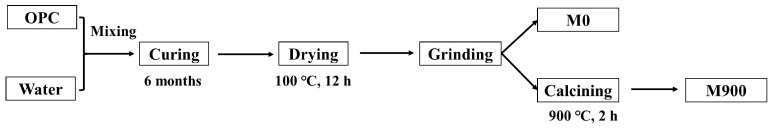
Flowchart of preparing simulated waste cement.

The Portland cement CEM I 42.5N, purchased from Fushun Cement Co., Ltd., Fushun, China, which has a high content of clinker, was used as the calcium and silicon source for the hydrothermal synthesis of xonotlite. As the amorphous SiO_2_ has high reactivity, the silica fume powder supplied by Shenmu Xinfuyuan Chemical Co. Ltd., Shenmu, China, was incorporated to supplement the silicon source for the synthesis. The chemical compositions of Portland cement and silica fume are shown in Table 1, and the mineral composition of Portland cement is shown in Table 2. The preparation of simulated waste cement is shown in Figure 1. The as-received cement was wet-mixed with a water-to-solid ratio (w/s) of 0.55 and cured in sealed polyethene bags at room temperature for 6 months, and the hydrated cement was employed to simulate the waste cement. After curing, the hydrated cement was dried at the temperature of 100 °C for 12 h to remove the free water and then ground to a powder with a particle size of less than 80 μm. As described above, heat treatment of the hydrated cement would enhance the reactivity of the hydrated cement. To clarify the effect of the reactivity of the hydrated cement on the hydrothermal synthesis of xonotlite, part of the dried hydrated cement powder (marked as M0) was directly used, and the other part was first calcined at 900 °C for 2 h (marked as M900) and then used for hydrothermal synthesis of xonotlite. Thus, the effect of the reactivity of the hydrated cement on the hydrothermal synthesis of xonotlite in this study was comparatively investigated by using M0 and M900 as the raw material.

### 2.2. Synthesis Procedure

**Figure 2:**
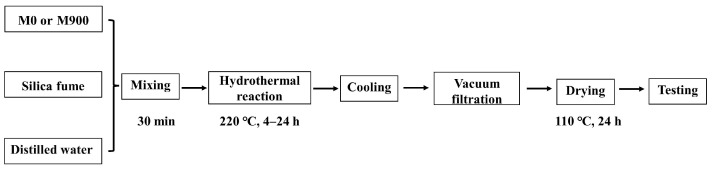
Flowchart of hydrothermal synthesis of xonotlite.

The hydrated cement (M0) or calcined hydrated cement (M900) was blended with silica fume as the corrective material at a Ca/Si molar ratio based on the composition of the ideal stoichiometric xonotlite (6CaO·6SiO_2_·H_2_O). The mixtures were dispersed in the distilled water with w/s of 30. The dispersion slurry was stirred for 30 min with the speed of 700 r/min by a digital display electric mixer (Jintan District Xicheng Xinrui instrument factory, Changzhou, China) at room temperature to make sure M0 (or M900) and silica fume were intensively mixed with water. After stirring, the slurry was transferred to a 5 L high-pressure reaction kettle (Weihai Huanyu Chemical Machinery Co. Ltd., Weihai, China) for hydrothermal synthesis at 220 °C (2.3 MPa) for different times (4, 8, 12, 24 h) at the stirring speed of 300 r/min. Finally, the slurry was filtered and dried at 110 °C for 24 h and then ground with mortar and pestle before further characterization. The experimental steps mentioned above are shown in Figure 2.

### 2.3. Characterization Methods

The hydrated cement (M0), calcined hydrated cement (M900) and hydrothermal products were ground into a powder with particle size less than 80 μm for phase characterization by X-ray diffraction (XRD, D8 advance, Bruker, Bremen, Germany) with 2θ ranging from 5° to 60°, using Cu Kα radiation (λ = 1.54056Å) at 40 kV and 30 mA with a scan speed of 5°/min.

The morphologies and structures of the above samples were detected with a scanning electron microscope (SEM, SIGMA HD, Zeiss, Gina, Germany) with an accelerating voltage of 5 kV.

Thermogravimetric sensing coupled with differential scanning calorimetry (TG-DSC) (NETZSCH STA 449F3) was employed to describe the decomposition process of the hydrated cement (M0) and the hydrothermal products. The rate of mass loss of M0 and hydration products per unit of time are expressed by calculating the first derivative of TG (DTG). The hydrated cement powder (M0) and hydrothermal products powder with the sample weight of 5.00 (±0.1) mg were heated at a rate of 10 °C/min over the temperature range of 30–1000 °C in a nitrogen atmosphere with the gas flowing at a velocity of 20 mL/min.

## 3. Results and Discussion

### 3.1. Characterization of Hydrated Cement without and with Heat Treatment

**Figure 3:**
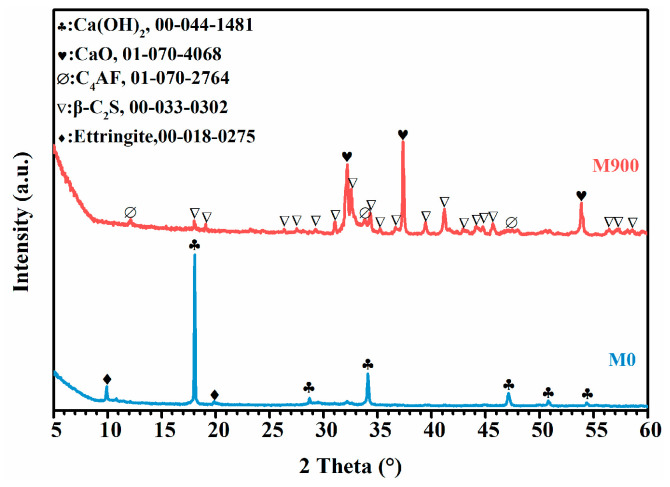
XRD patterns of hydrated cement M0 and M900.

The XRD patterns of hydrated cement powder without heat treatment (M0) and with calcination at 900 °C (M900) are shown in Figure 3. It is seen in the figure that the strong peaks of portlandite (CH, Ca(OH)_2_) and weak peaks of ettringite (AFt, 3CaO·Al_2_O_3_·3CaSO_4_·32H_2_O) were detected for Sample M0. It should be noticed that the main hydration product, C-S-H gel, cannot be detected by XRD analysis, which we attribute to its poor crystallinity [28]. After calcination at 900 °C, the XRD patterns indicate that lime (CaO) and belite (β-C_2_S, β-2CaO·SiO_2_) become the dominant crystal phases, and ferrite (C_4_AF, 4CaO·Al_2_O_3_·Fe_2_O_3_) appears as a minor phase in sample M900. The peaks of portlandite and ettringite vanished. It clearly seems that CaO was generated mostly from the dehydration of CH, and β-C_2_S came from the decomposition of C-S-H gel during the calcination [29,30].

**Figure 4:**
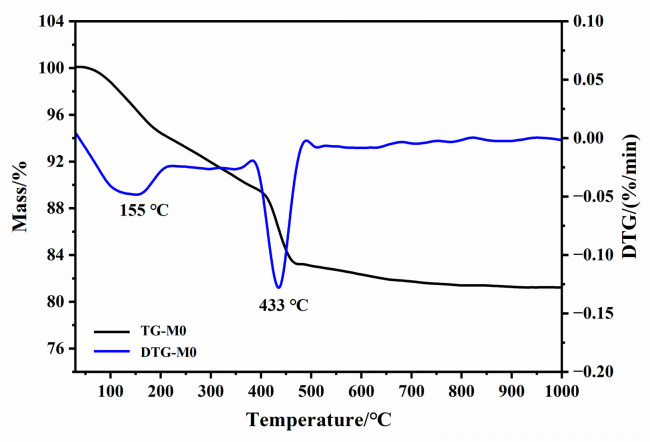
TG-DSG curves of hydrated cement M0.

The thermal analysis (TG-DTG) of hydrated cement powder M0 is presented in Figure 4. The DTG curve demonstrates that there are three ranges of heating temperature that correspond to the relatively sharp decrease in weight, including 50–200 °C, 380–480 °C and 590–680 °C. The weight loss from 50 to 200 °C was around 5.11%, resulting mostly from the escape of the residual absorbed water and the dehydration of AFt. The decomposition of CH occurred from 380 °C to 480 °C, accompanied by 5.04% weight loss. The moderate loss of gravity at around 624 °C was deduced to be the decarbonation of CaCO_3_, which might be generated during the drying and/or sample preparation period. Therefore, the CaO in M900 (Figure 3) is produced mostly by the dehydration of Ca(OH)_2_ and rarely from the decarbonation of CaCO_3_. The dehydration of C-S-H gel might cover a wide range of heating temperatures. The research of Matthew et al. stated that the crucial loss of water occurs from 105 °C to 300 °C [31]. Wang et al. and Bogas et al. found that there would be a further loss of chemically bound water of C-S-H gel when the heating temperature is elevated up to 750 °C or 850 °C [8,15]. The generation of β-C_2_S is reported to be attributed to the decomposition of the C-S-H gel [29,30,32]. These results also demonstrate that CaO is generated mostly from the dehydration of CH and β-C_2_S comes from the decomposition of C-S-H gel during the calcination.

**Figure 5:**
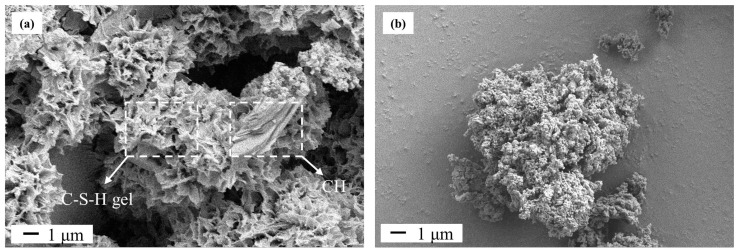
SEM images of hydrated cement M0 (**a**) and after calcination M900 (**b**).

SEM images of the as-hydrated cement (M0) and calcined hydrated cement (M900) are shown in Figure 5. The honeycomb structure in Sample M0 (Figure 5a) corresponds to the dried C-S-H gel, and the layered plates match up with the hydration product CH [33], which are produced by the reaction of C_3_S and C_2_S in Portland cement with water. The microstructure of calcined hydrated cement (M900) is mainly grain-shaped, which is quite different from M0. This result is because the C-S-H gel loses chemical water, causing the layer spacing to decrease. As the calcination temperature increased, Ca(OH)_2_ decomposed into CaO, and C-S-H gel began to decompose into C_2_S. At 900 °C, C-S-H gel almost completely decomposed into C_2_S (β-C_2_S) with higher crystallinity [30,32]. It has been reported [32] that as the calcination temperature of hydrated cement increased, the particles gradually split, and only small spherical particles could be observed at 900 °C. It can be seen in Figure 5 that the size of the particles in calcined hydrated cement (M900) (Figure 5b) was less than 100 nm, which is much smaller than those in the hydrated cement (M0) (Figure 5a).

### 3.2. Analysis of Hydrothermal Synthesis Products

**Figure 6:**
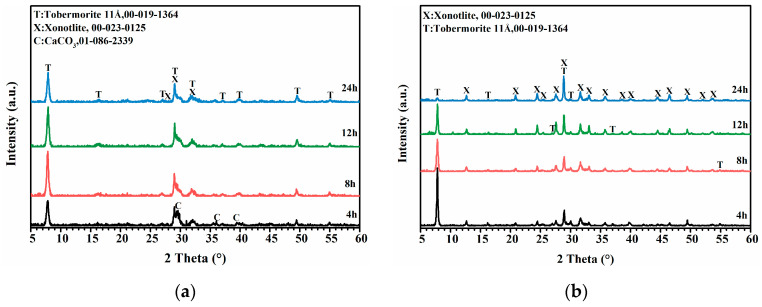
XRD patterns of samples synthesized at 220 °C hydrated cement M0 (**a**) and calcined hydrated cement M900 (**b**).

The XRD patterns in Figure 6a,b compare the phase evolution of hydrothermal synthesis products using hydrated cement (M0) and calcined hydrated cement (M900). At the reaction time of 4 h, the strong peak of 11 Å tobermorite and very weak peaks of xonotlite were detected in the samples synthesized from M0 and M900 (Figure 6a,b). With the extension of reaction time to 12 h, the peaks of 11 Å tobermorite synthesized from M0 slightly increased (Figure 6a), and the peak of 11 Å tobermorite synthesized from M900 decreased at the same time, and the xonotlite peaks increased to a very small extent (Figure 6b).

However, when the reaction time is extended to 24 h, the major phases of sample synthesis using M0 and M900 are quite different. The main phase of the product synthesized from M0 was still 11 Å tobermorite, and the peak strength of xonotlite increased slowly (Figure 6a). In contrast, in the sample synthesized using M900, the peak of 11 Å tobermorite at 2θ value of 7.81° almost disappeared, and the strong peaks of xonotlite were detected (Figure 6b).

It can be seen in Figure 6 that in the early stage of the hydrothermal reaction (less than 12 h), utilizing hydrated cement (M0) or calcinated hydrated cement (M900) has no obvious influence on the phase composition of hydration products. However, with the prolonging of hydrothermal synthesis duration to 24 h, in comparison with hydrated cement (M0), the calcined hydrated cement (M900) significantly promotes the formation of xonotlite. The above results are different from the study of Kuzielová et al. [34]. This study shows the product synthesized from the silica fume-Portland cement system by static autoclave method does not contain xonotlite. This difference may relate to the effective Ca/Si ratio and ion transport rate in the reaction process.

In this work, as seen in Figure 5, the calcined hydrated cement (M900) (Figure 5b) had a much smaller particle size than hydrated cement (M0) (Figure 5a), and consequently M900 must have a higher dissolution rate than M0 when in contact with water [35]. Therefore, the reactivity of CaO and β-C_2_S in calcined hydrated cement (M900) should be much higher than those of C-S-H gel and Ca(OH)_2_ in hydrated cement (M0), so a lot of Ca^2+^ ions can be easily dissolved to react with [SiO_4_]^4−^. In this case, a large amount of transition phase (11 Å tobermorite) was generated in the initial synthesizing period, which could convert into xonotlite when increasing the duration of hydrothermal treatment to 24 h (Figure 6b).

**Figure 7:**
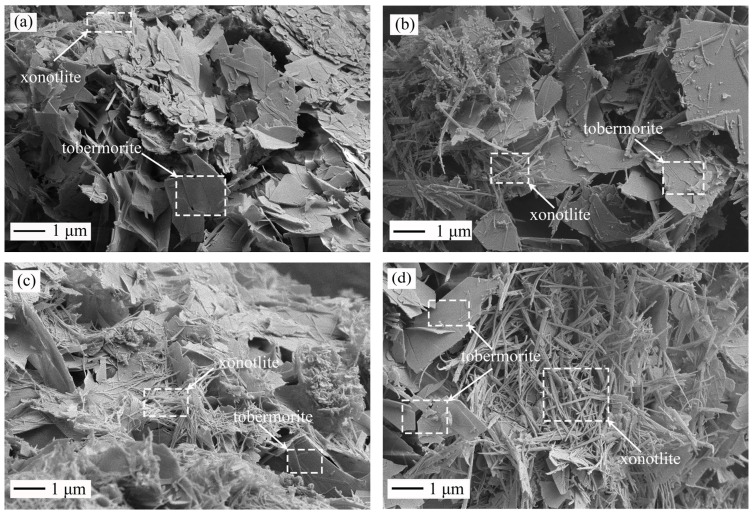
SEM images of the products were synthesized from hydrated cement (M0) and calcined hydrated cement (M900) at 220 °C. Note: (**a**) M0-4 h, (**b**) M900-4 h, (**c**) M0-8 h, and (**d**) M900-8 h.

**Figure 8:**
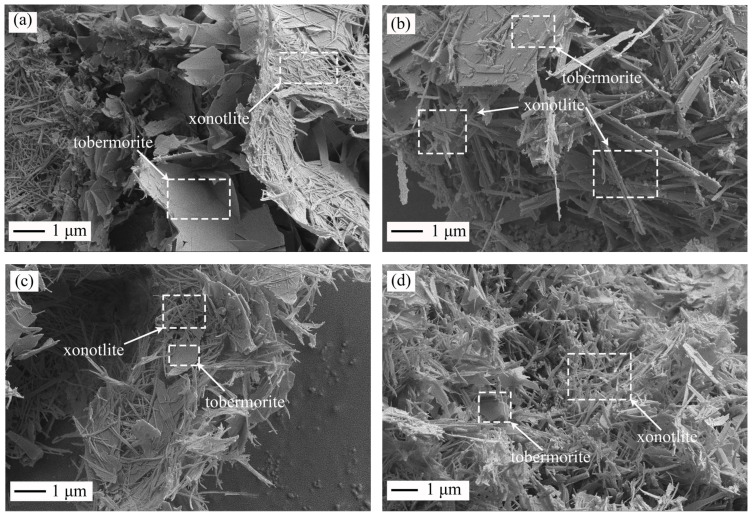
SEM images of the synthesis products from hydrated cement (M0) and calcined hydrated cement (M900) at 220 °C. Note: (**a**) M0-12 h, (**b**) M900-12 h, (**c**) M0-24 h, and (**d**) M900-24 h.

It is evident from Figure 7 that the major plate phase corresponding to 11 Å tobermorite and the minor fiber-like xonotlite were similar in the hydrothermal product using M0 (Figure 7a) and using M900 (Figure 7b) [36]. This phenomenon is because, at the beginning of the reaction, Ca^2+^ and SiO_4_^4−^ in solution react to form a poorly crystallized C–S–H gel that has good periodicity parallel to the ab plane but poor periodicity in the c direction. As the reaction went on, the CaO layers of the C–S–H gel and silicate chains became more ordered, having increasing periodicity in the 001 direction to form the complete plate-like 11 Å tobermorite [37]. As the reaction continued, between 8 h (Figure 7) and 12 h (Figure 8), there was no obvious difference in the amount of major flake-like 11 Å tobermorite and minor fine fibrous xonotlite, no matter whether M0 or M900 was used in the starting materials. These results indicate that at the early stage of the reaction, there is little difference in the phase composition of the products synthesized by using hydrated cement (M0) and calcined cement (M900), which is consistent with the XRD results (Figure 6).

With the synthesis time increasing to 24 h, it can be seen that the morphologies of the samples synthesized by M0 (Figure 8c) and M900 (Figure 8d) exhibited a remarkable difference. The morphology of 11 Å tobermorite synthesized from M0 changed from plates to small sheets, and the needle-like xonotlite had no obvious change (Figure 8c). In comparison, in the sample synthesized by M900, massive grass-like xonotlite and a small amount of sheet-shaped 11 Å tobermorite were observed (Figure 8d), which may have been due to the interlayer water of 11 Å tobermorite dissociation into OH^−^ and attracted free Ca^2+^, and the [SiO_4_]^4−^ three-link chain on both sides of the CaO layer condensed into a [Si_6_O_17_]^10−^ double chain [38,39], which resulted in the CaO layer combining with Si–OH in tobermorite phase and the Ca-O bond of CaO layer acting as a connection between the double chain. Therefore, the 11 Å tobermorite transformed into fiber-like xonotlite. This result is similar to the study of Zhou et al. [40]: when the effective Ca/Si ratio was higher than 0.83, it was difficult for 11 Å tobermorite to remain stable at the higher reaction temperature.

The above results are consistent with those of XRD (Figure 6). Compared to the variations in morphology of the samples in Figure 7 and Figure 8, it can be found that when the reaction time is 4–12 h, the phase composition and morphology of the hydrothermal products synthesized by M0 and M900 differed little. However, when the reaction time was 24 h, the samples synthesized by M900 were mainly fibrous xonotlite, and the samples synthesized by M0 were mainly flake tobermorite.

According to the above results (Figure 6, Figure 7 and Figure 8), it can be seen that after 24 h of hydrothermal synthesizing, the phase compositions and microstructures of the products using calcined hydrated cement (M900) and hydrated cement (M0) as initial raw materials were quite different.

For calcined hydrated cement (M900), the β-C_2_S in M900 was of low crystallinity, owing to the relative calcination temperature of 900 °C, rather than that of the β-C_2_S in OPC clinkers [30]. As a result, the β-C_2_S in M900 could quickly react with water to produce C-S-H gel and CH, and the CaO in M900 could also react with water to form CH, resulting in more Ca^2+^ to dissolve in the water [32]. Therefore, when M900 is used as the starting material, more Ca^2+^ could be dissolved in the solution to react with [SiO_4_]^4−^. With the extension of reaction time (24 h), the effective Ca/Si ratio in the solution nears one [41], and a large amount of transitional phase 11 Å tobermorite generated at the earlier stage (12 h) could convert into xonotlite at the late stage (24 h) of the reaction.

As for the hydrated cement (M0), the 6 months of curing led to the C-S-H gel and CH in M0 having higher crystallinity and lower chemical reactivity when reacting with water, which may require higher initial reaction temperature and reaction time to destroy its structure and dissolve Ca^2+^. Therefore, the dissolution rate of Ca^2+^ and the rate of reaction with [SiO_4_]^4−^ are very slow, leading to the effective calcium–silicon ratio in the solution being hardly close to one (less than one) [41] at the late stage of hydrothermal reaction (24 h). Hence, the transition phase 11 Å tobermorite synthesize at the early stage (12 h) is difficult to convert into xonotlite at the late stage (24 h) of hydrothermal reaction. The above results can be summarized that the reactivity of starting materials affects the major phase of the end product: the main phase of the sample synthesized from hydrated cement (M0) for 24 h is 11 Å tobermorite, and the major phase of the sample synthesized from calcined hydrated cement (M900) under the same conditions is xonotlite.

A previous study [38] in this system showed that the reaction can produce a mixture of 11 Å tobermorite and xonotlite, and the two phases have very similar diffraction patterns due to many overlapping peaks. The relative number of these phases varied over the course of the experiment. As a result, quantifying the amount of each component at any one time is extremely difficult. Therefore, in this experiment, TG-DTG was used to estimate and compare the content of xonotlite in hydrothermal products synthesis from M0 and M900 in the same reaction time.

**Figure 9:**
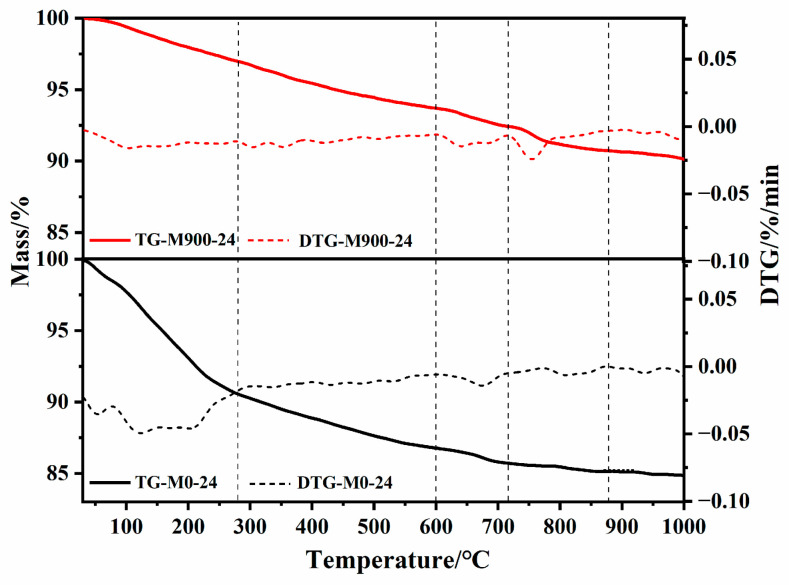
The TG-DTG curves of samples synthesized from hydrated cement (M0) and calcined hydrated cement (M900) at 220 °C for 24 h.

Figure 9 shows the TG-DTG curves of products synthesized from hydrated cement (M0) and calcined hydrated cement (M900) at 220 °C for 24 h. It can be seen that the weight loss from 30 °C to 280 °C was caused by the dehydration of free water and crystal water in 11 Å tobermorite [42], which was accompanied by 9.40% weight loss (M0-24) and 3.10% weight loss (M900-24). The weight loss by dihydroxylation of tobermorite occurred from 600 to 715 °C, accompanied by 1.17% (M0-24) and 1.31% of weight loss (M900-24). In the temperature range of 715–880 °C, the weight loss of hydrothermal synthesis product was caused by the dihydroxylation of xonotlite [43], and the weight loss of the product synthesized by M900 (1.86%) was higher than that of M0 (0.45%), from which it can be calculated that the product synthesized by M900 had a higher content of xonotlite (74%) than the product synthesized by M0 (18%). This result further indicates that calcination improves the reactivity of calcium and silicon in hydrated cement, which promotes the synthesis of xonotlite.

## 4. Conclusions

In this study, Portland cement hydrated for 6 months was prepared to simulate waste cement, and the reactivity of calcium and silicon in hydrated cement was improved by calcination at 900 °C. The reactivity of calcium and silicon in hydrated cement during the hydrothermal synthesis of xonotlite was studied for the first time. The results can be summarized as follows.

(1) C-S-H gel and CH are the two main phases in hydrated cement. After calcination, CaO and β-C_2_S are produced from the decomposition of CH and C-S-H gel, which have finer particles. Consequently, these dehydrated phases (CaO and β-C_2_S) can rapidly form a new CH and C-S-H gel when coming into contact with water. Therefore, the new CH and C-S-H gel generated from the calcined cement (M900) have higher reactivity than those in the hydrated cement (M0).
(2) At the late stage of hydrothermal reaction (24 h), the effective Ca/Si ratio in the solution with hydrated cement (M0) is not close to one (less than one), and the radio in the solution with calcined hydrated cement (M900) is close to one. As a result, the 11 Å tobermorite synthesized at the early stage (12 h) was converted into xonotlite at the late stage (24 h) of hydrothermal reaction in the sample with M900 and remained unchanged in the sample with M0.
(3) The products synthesized from hydrated cement contained large amounts of 11 Å tobermorite and small amounts of xonotlite at the hydrothermal temperature of 220 °C. Under the same reaction conditions, the proportion of xonotlite in the products synthesized by calcined hydrated cement increased from 18% (synthesized from hydrated cement) to 74% (synthesized from calcined hydrated cement) when the reaction time reach 24 hed.

## Figures and Tables

**Table 1:** Oxide compositions of the Portland cement CEM I 42.5 N and silica fume.

Sample	Oxide Composition (Mass%)									
	CaO	SiO_2_	Al_2_O_3_	Fe_2_O_3_	SO_3_	Na_2_O	MgO	P_2_O_5_	K_2_O	Cr_2_O_3_
OPC	64.03	21.14	4.69	3.15	2.09	0.56	2.57	0.00	0.00	0.00
SF	0.36	96.62	0.60	0.14	2.09	0.44	0.19	0.07	0.63	0.05

**Table 2:** Mineral composition of Portland cement CEM I 42.5 N given by the producer.

Sample	Mineral Composition (Mass%)						
	C_3_S	C_2_S	C_3_A	C_4_AF	f-CaO	MgO	SO_3_
OPC	58.34	19.32	7.13	10.00	0.98	0.63	0.68

## Data Availability

All the data are available within the manuscript.
